# Acute Bilateral Salter-Harris II Distal Radii Fractures in a Skeletally Immature Athlete

**DOI:** 10.7759/cureus.36994

**Published:** 2023-04-01

**Authors:** Pasquale Gencarelli, Rahul Mittal, James M Lee

**Affiliations:** 1 Orthopaedic Surgery, Rutgers Robert Wood Johnson Medical School, New Brunswick, USA; 2 Health Informatics, Rutgers University, Newark, USA; 3 Orthopaedic Surgery, Orange Orthopaedic Associates, West Orange, USA

**Keywords:** conservative treatment, salter harris type 2, youth athlete, skeletally immature, bilateral distal radii fractures

## Abstract

Distal radius fractures are a common location of physeal injuries in skeletally immature adolescents. However, reports of athletics-related acute bilateral distal radius physeal injuries are rare. Therefore, there is a need for further literature to demonstrate both the early recognition and prevention of these injuries to ensure young athletes are able to safely train and compete. We present the case of acute bilateral Salter-Harris II distal radii fractures in a 14-year-old athlete during participation in a high-energy impact sport.

## Introduction

Competitive athletics and weightlifting have become increasingly prominent in the lives of young athletes over the last several decades. However, in skeletally immature individuals, the physis is three to five times weaker than connective tissue and vulnerable to injury under tension and shear stress, resulting in possible irreversible damage to the active physis [1,2]. In particular, distal radius fractures are a common location of physeal injuries in skeletally immature adolescents, accounting for roughly 15-20% of injuries, and are usually the result of low-energy mechanisms, such as falls, but rarely have associated injuries [3]. Moreover, the distal radius physis does not fuse until 16-18 years of age, leaving a large time frame for a growth plate injury to occur [4]. Yet, despite these concerns, reports of acute athletic-related bilateral distal radii physeal injuries are rare [5,6]. Only a handful of case reports exist in the literature describing sports or weightlifting-related acute bilateral distal radii physeal injuries and their management in skeletally immature athletes [7-10]. Thus, there is a need for further literature to demonstrate both the early recognition and prevention of these injuries to ensure young athletes are able to safely train and compete. We present a case of bilateral Salter-Harris II distal radii fractures in a 14-year-old athlete during participation in a high-energy impact sport.

## Case presentation

A 14-year-old male athlete with no past medical or surgical history presented to the emergency department with complaints of bilateral wrist pain, swelling, and visible deformities. The patient reported he was participating in a football game when he fell backward onto his bilateral outstretched hands. The patient reported immediate pain as well as continued swelling after the fall and came to the emergency department several hours after the injury. The patient denied current medication use or known drug allergies. The patient denied current or previous alcohol, tobacco, or illicit drug use. Family history was noncontributory.

**Figure 1:**
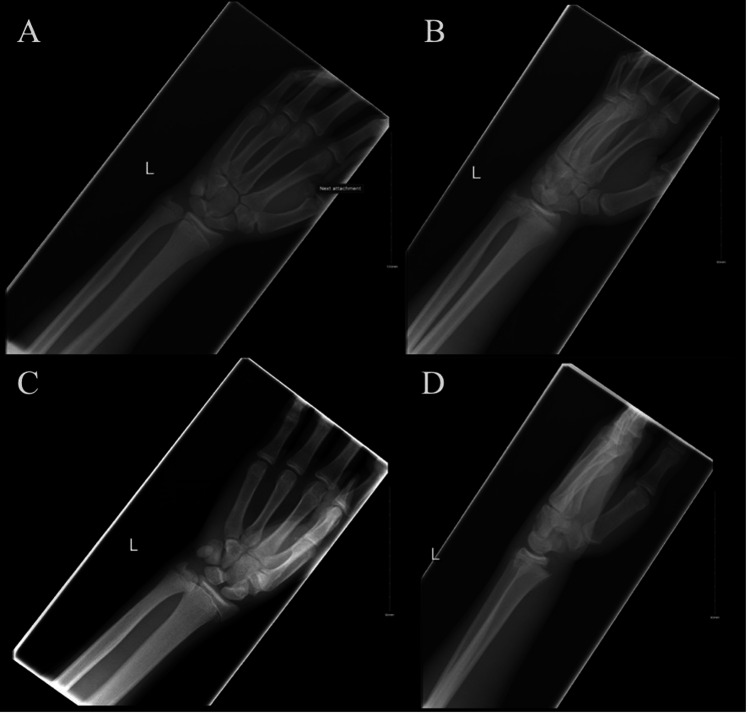
Left wrist radiographs pre-reduction Postero-anterior (A), oblique (B and C), and lateral (D) radiographs of the left wrist pre-reduction demonstrating left Salter-Harris II distal radius fracture with dorsal angulation.

**Figure 2:**
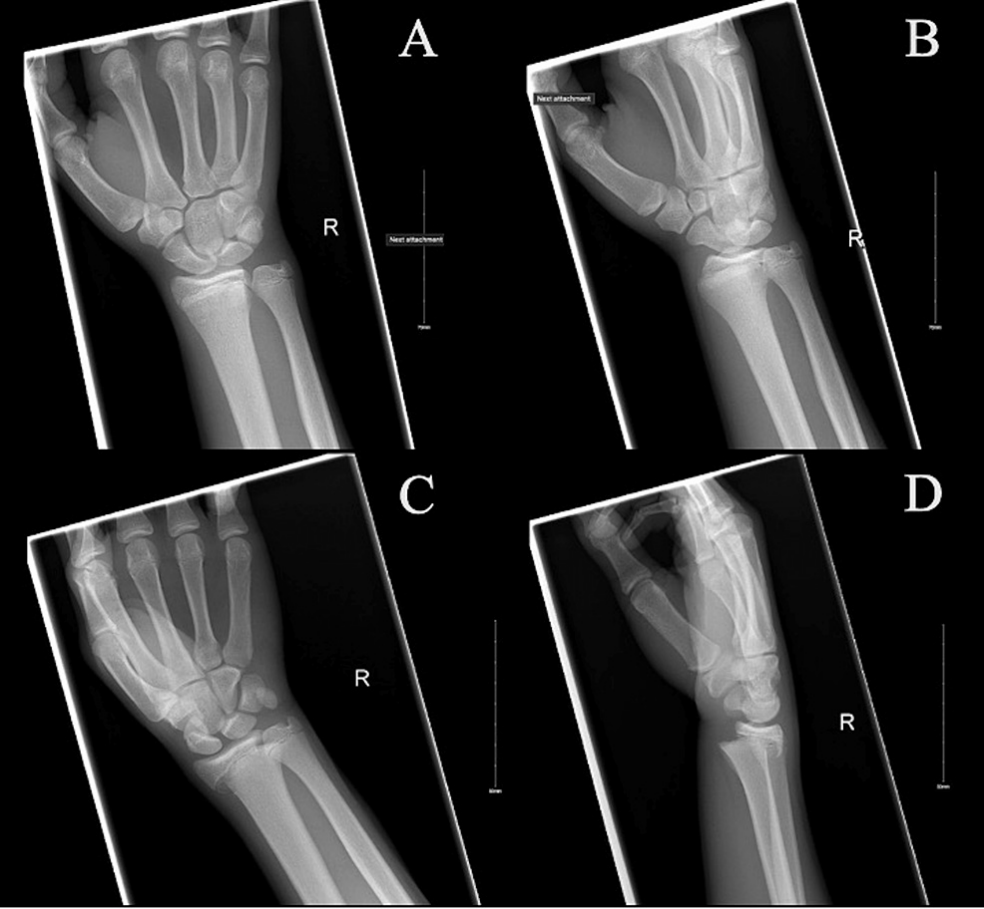
Right wrist radiographs pre-reduction Postero-anterior (A), oblique (B and C), and lateral (D) radiographs of right wrist pre-reduction demonstrating a right Salter-Harris II distal radius fracture with dorsal angulation.

Physical exam revealed bilateral “dinner-fork” deformities and effusions of the bilateral wrists, however, all compartments were soft to palpation. The patient’s range of motion was limited secondary to pain in flexion and extension as well as an ulnar and radial deviation of the bilateral wrists. The patient was neurovascularly intact bilaterally with no open fractures observed. Bilateral radiographs were obtained and demonstrated dorsally displaced Salter-Harris II fractures of the bilateral distal radii which are depicted in Figures 1A-1D and Figures 2A-2D.

**Figure 3:**
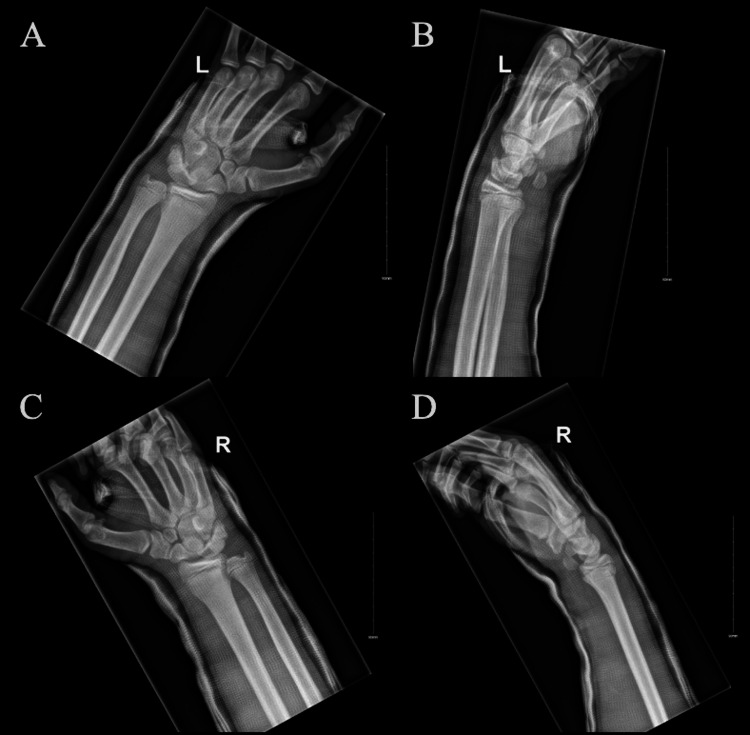
Bilateral wrist radiographs post-reduction Post-closed reduction and short arm casting of bilateral wrists; Posteroanterior (A) and lateral (B) radiographs of the left wrist and posteroanterior (C) and lateral (D) radiographs of the right wrist

No labs were ordered. Prior to attempting a closed reduction of both wrists in the emergency department, standard parental consent was obtained in line with hospital policy. The patient received a hematoma block of both wrists using 10 mL of 1% lidocaine, was closed reduced with the aid of a mini C-arm, and placed in bilateral short arm casts using fiberglass and Webril, which were both molded to resist apex volar angulation of the bilateral fractures. Post-reduction radiographs in the emergency department demonstrated acceptable length, alignment, and rotation (Figures 3A-3D). Follow-up outpatient radiographs 10 days after initial reduction demonstrated no displacement of the bilateral fractures.

**Figure 4:**
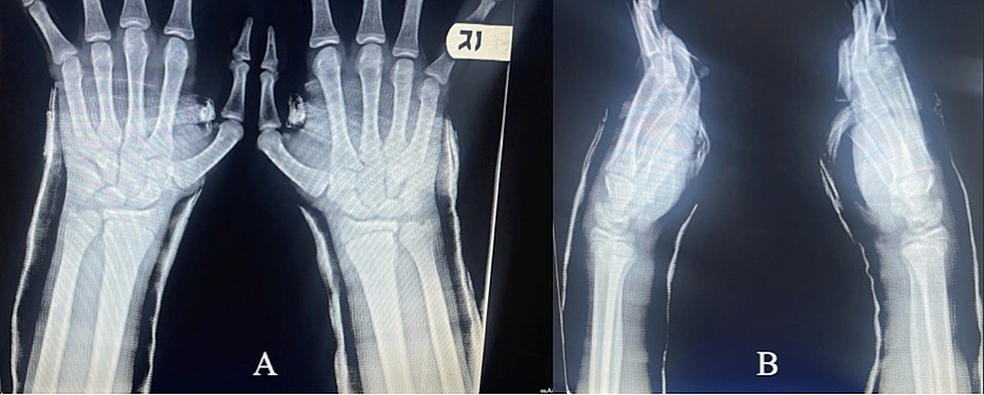
Bilateral wrist radiographs five and a half weeks post-injury Posteroanterior (A) and lateral (B) radiographs of the left and right wrists immediately prior to short arm cast removal at five and half weeks post-injury demonstrating interval healing of the bilateral distal radii fractures

The patient remained non-weight bearing in the bilateral upper extremities for five and a half weeks, at which time radiographs demonstrated healing of the bilateral Salter-Harris II distal radii fractures with acceptable length, alignment, and rotation (Figures 4A, 4B). At three months post-injury, the patient was cleared to return to play with no restrictions.

## Discussion

The participation of skeletally immature athletes in competitive sports and weightlifting has continued to increase. However, the physes of skeletally immature individuals are fragile and vulnerable to injury under tension or shear stress [1,2]. Specifically, the distal radius epiphysis does not fuse until ages 16-18 years old, leaving most young athletes vulnerable to physeal injury and possible early growth arrest or angular deformity [4,5]. As a result, increased awareness and guidance on the prevention and treatment of these potentially devastating injuries are required.

Only a handful of case reports exist reporting acute bilateral distal radii physeal fractures related to sports or weightlifting (Table 1) [7-10]. Four studies representing six patients (aged 13-17; all males), reported bilateral distal radii physeal fractures (Salter-Harris I or II) [7-10]. Of the four studies, all patients were performing either overhead military shoulder press [7-9] or overhead barbell press during the clean and jerk at the time of injury [10]. All six patients were treated with closed reduction and casting, however, only two studies reported follow-up outcomes on patients [9,10]. Gumbs et al. did not report on the specific follow-up time, however, the authors reported the two male patients’ “healing was uneventful” [9]. Ercan et al. reported on one male patient and noted complete fracture healing at three months, full return to training participation at six months, and no reinjuries at one year [10]. Common to all patients was hyperextension of the wrists at the time of injury resulting in Salter-Harris I or II fractures.

**Table 1:** Case reports on sports and weightlifting related to acute bilateral distal radii physeal injuries in skeletally immature athletes M = male; * = one patient had a right Salter-Harris I distal radius fracture

Study	Number of cases	Age (years)	Injury location	Salter-Harris classification	Activity	Outcome
Ryan et al. [7]	5 M	14-17	Unilateral distal radius (bilateral n=2)	II (right, left)	Weightlifting	Follow-up not reported
Jenkins et al. [8]	1 M	13	Bilateral distal radii	II (right, left)	Football	Follow-up not reported
Gumbs et al. [9]	2 M	12, 14	Bilateral distal radii	II (right, left)*	Weightlifting	Follow-up time not reported; “healing was uneventful”
Ercan et al. [10]	1 M	16	Bilateral distal radii	I (right), II (left)	Weightlifting	Healed fractures at three months, return to play at six months, no complications at one year

Our patient had a similar mechanism of injury as the forward momentum if his body weight was transferred through his bilateral hyperextended wrists after falling on his outstretched hands. In skeletally immature individuals, the peak incidence of distal radius fractures is 12-14 years old in boys and 10-12 years old in girls related to the decreased level of skeletal mineralization and density that exists during growth spurts [6]. As a result, most physeal fractures occur within the hypertrophic zone at the junction of calcified and uncalcified hypertrophic cells [6]. Moreover, an injury that can cause a sprain in an adult is also enough force to cause a serious growth plate injury in skeletally immature individuals [6]. These underlying principles, coupled with our patient’s mechanism of injury, likely explain the cause of his bilateral distal radii physeal fractures. Thus, although rare injuries, we recommend young athletes, trainers, coaches, and parents maintain a high index of suspicion for distal radius physeal injuries and seek prompt care when injuries are suspected, especially in athletes within the pubertal growth spurt timeframe. Thankfully, for nondisplaced distal radius Salter-Harris I or II injuries with the potential for growth and remodeling, most patients recover with non-operative management and have no complications such as in our patient.

Moreover, we acknowledge the benefits of young athlete participation in physical activity, such as sports and weightlifting, largely outweigh the risks of associated injuries. However, we believe preventive measures can be implemented to minimize the risk of physeal injury in young athletes, especially for those experiencing periods of rapid growth. First, coaches should cycle training loads, with well-defined rest periods, and use a variety of drills during practice to reduce stress-related physeal injuries and avoid overtraining. Moreover, to minimize acute physeal injuries for collision sports (i.e. football), factors such as physical maturity as well as fitness and skill level should be considered to equalize competition among peers of similar chronological age. In addition, when weightlifting, athletes should avoid exercises that put excessive stress on the physis such as hyperextension of the wrists with an overhead military press, clean and jerk, or supine barbell bench press. Lastly, young athletes should train with high repetitions and low weight, utilize spotters and proper form, and weightlift under the supervision of trained personnel to minimize injury. Future research should focus on the epidemiology and associated risk factors of physeal injuries in young athletes. Specifically, the rate of physeal injury during the pubertal growth spurt, the correlation of the type of physeal fracture with the mechanism of injury, and the possible reduction of physeal injuries in athletes with cycled training loads should be elucidated.

## Conclusions

With the increasing participation of young athletes in competitive athletics and weightlifting, athletes, parents, and coaches should maintain a high index of suspicion for physeal injuries, especially during times of rapid growth. Our case highlights the fragility of the growth plates during the pubertal window and the rare possibility of bilateral distal radii physeal injuries during high-energy impact sports. Future research should focus on the epidemiology and associated risk factors of physeal injuries to better guide prevention strategies.
